# The Postoperative Paradoxical Septum (POPS): A Comprehensive Review on Physio-Pathological Mechanisms

**DOI:** 10.3390/jcm13082309

**Published:** 2024-04-17

**Authors:** Emanuele Di Virgilio, Paolo Basile, Maria Cristina Carella, Francesco Monitillo, Daniela Santoro, Michele Davide Latorre, Silvia D’Alessandro, Laura Fusini, Fabio Fazzari, Gianluca Pontone, Andrea Igoren Guaricci

**Affiliations:** 1 Cardiology Unit, Hospital of Corato, ASL Bari, 70033 Corato, Italy; emanueledrit@gmail.com; 2 University Cardiology Unit, Interdisciplinary Department of Medicine, “Aldo Moro” University School of Medicine, AOUC Polyclinic, 70121 Bari, Italy; paolo.basile@uniba.it (P.B.); m.c.carella92@gmail.com (M.C.C.); dr.francescomonitillo@gmail.com (F.M.); danina2012@gmail.com (D.S.); latorre.michele.d@gmail.com (M.D.L.); 3 Neurology Unit, Hospital of Altamura, 70022 Altamura, Italy; 4 Department of Perioperative Cardiology and Cardiovascular Imaging, Centro Cardiologico Monzino IRCCS, 20138 Milan, Italy; laura.fusini@cardiologicomonzino.it (L.F.); fabio.fazzari@cardiologicomonzino.it (F.F.); gianluca.pontone@cardiologicomonzino.it (G.P.); 5 Department of Biomedical, Surgical and Dental Sciences, University of Milan, 20122 Milan, Italy

**Keywords:** postoperative paradoxical septum, abnormal septal motion, cardiac imaging

## Abstract

The interventricular septum (IVS) is a core myocardial structure involved in biventricular coupling and performance. Physiologically, during systole, it moves symmetrically toward the center of the left ventricle (LV) and opposite during diastole. Several pathological conditions produce a reversal or paradoxical septal motion, such as after uncomplicated cardiac surgery (CS). The postoperative paradoxical septum (POPS) was observed in a high rate of cases, representing a *unicum* in the panorama of paradoxical septa as it does not induce significant ventricular morpho-functional alterations nor negative clinical impact. Although it was previously considered a postoperative event, evidence suggests that it might also appear during surgery and gradually resolve over time. The mechanism behind this phenomenon is still debated. In this article, we will provide a comprehensive review of the various theories generated over the past fifty years to explain its pathological basis. Finally, we will attempt to give a heuristic interpretation of the biventricular postoperative motion pattern based on the switch of the ventricular anchor points.

## 1. Introduction

Following cardiac surgery (CS), patients often exhibit a new abnormal motion of the interventricular septum (IVS) during echocardiographic follow-up. This issue has been reported since the early 1970s [1,2,3,4,5]. Although other imaging techniques can detect it, echocardiography remains the most common method [6,7,8,9,10,11]. This abnormal septal motion is known by different names, such as reversed septal movement, pseudo-paradoxical septum, or simply abnormal septal motion (ASM). In this review, we will use “ASM” to indicate generic alteration of septal movement and “postoperative paradoxical septum” (POPS) for specific postoperative septal kinetic alterations. The definition of POPS has evolved with technology advancements and imaging techniques. It can be comprehensively defined as a new-onset postoperative non-respirophasic flat or centrifugal systolic motion of the IVS with normal septal wall thickening, preserved left ventricular geometry, unchanged global systolic function, and normal septal perfusion and metabolism. POPS represents a *unicum* in the panorama of paradoxical septa as it does not induce significant ventricular morpho-functional alterations nor negative clinical impact. However, a dyskinetic IVS can be observed in various pathological conditions, such as myocardial ischemia or necrosis, intraventricular conduction delays, pre-excitation, constriction physiology, pericardial effusion, right heart overload, or congenital absence of the pericardium (CAP) [12,13,14,15,16]. Some of these conditions can complicate the postoperative period [10,17,18,19]. Furthermore, changes in the lateral wall can also occur, complicating the overall assessment of the postoperative systolic function of the left ventricle (LV) [20,21,22,23].

The pathophysiological basis of postoperative septal behavior is still debated, and several theories have been proposed over the years, including intraoperative myocardial damage and altered wall synchrony. However, current theories focus on extrinsic factors related to increased heart mobility and new anatomical relationships with the thoracic walls during the cardiac cycle [3,20,21,24]. Postoperative changes in the morphological and systolic function patterns of the right ventricle (RV) are also believed to play a pathogenetic role [25,26,27,28]. In this article, we provide a comprehensive review of the current state-of-the-art knowledge about POPS, tracing its essential stages from the initial observations to the most recent developments.

## 2. Epidemiology

A paradoxical septal bouncing is observed in a relatively large number of cases during the postoperative period. The reported incidence ranges from 29% to 100% in the early perioperative follow-up. Most studies have primarily focused on male adult or elderly patients undergoing various types of CS. Notably, a significant number of studies have specifically investigated coronary artery bypass graft surgery (CABG) [29], while there is limited data available on other types of cardiac interventions (Table 1).

In the largest population examined, consisting of 3292 cases, an overall POPS incidence of 39% was reported. Valvular heart surgery (VHS), particularly mitral valve surgery (MVS), showed the highest rate of POPS (60% of cases). Multivariate analysis indicated that the development of POPS was independently associated with the type of surgical approach, patient’s age, and cross-clamp time (*p* < 0.001), while gender had no significant impact [9]. A cohort of 256 subjects undergoing VHS had a similar overall incidence, although aortic valve surgery (AVS) had a significantly higher POPS incidence than MVS (64% vs. 36%, *p* < 0.01) [30]. Other authors also reported no statistically significant differences in the POPS incidence between CABG and VHS. They also reported a notable improvement in septal dyskinesia after a 12-month follow-up [31]. In a prospective study of 165 patients, the type of surgery and approach did not influence the development of POPS, with a decrease in prevalence from 73% to 25% during the late follow-up [24]. Similarly, previous studies based on small cohorts in the 70s and 80s consistently described a progressive POPS resolution during follow-up [3,20,32].

In contrast, Okada et al. reported the presence of POPS in all subjects during peri-operative control and in the late follow-up using gated blood-pool scans with technetium-99m-pyrophosphate (Tc-99m-PYP-GBPS) [33]. Moreover, using rest-gated single photon emission computed tomography (SPECT), POPS was observed in over two-thirds of the subjects during routine 2-year imaging follow-up after CABG [22]. Patients undergoing AVS showed similar findings after 19 months of follow-up using radionuclide angiocardiography (RNA) [34]. Therefore, the choice of imaging tool may affect the assessment of septal kinesis. However, there is a lack of comparative data between echocardiography and other imaging techniques. For example, in a qualitative assessment using Nuclear Magnetic Resonance (NMR), the presence of POPS was observed after 3 months from CABG, with different results reported between the echocardiographic evaluation and NMR [26]. De Nardo et al. found consistent results evaluating 34 patients after 10 days of uncomplicated CABG, with both echocardiography and RNA showing POPS, although RNA detected a higher prevalence. They also highlighted a high rate of postoperative increase in segmental ejection fraction in the posterolateral wall using RNA, without significant differences in the systolic thickening fraction of the posterior wall (PW) examined by echo-Motion-Mode (M-mode) [35]. It seems that techniques other than ultrasound tend to overestimate wall displacement when compared to echocardiography. This difference could be due to the varying definitions of POPS used. Ultrasound techniques often use quantitative assessment, while other methods rely on a qualitative approach. However, recent echocardiographic studies have reported a high prevalence of POPS using both qualitative and quantitative approaches during late follow-up [36].

It is worth noting that the method of approach can impact the diagnosis of POPS, even when using the same imaging tool. Lehman et al. discovered that the incidence of POPS was 100% and 76% when using qualitative and quantitative approaches, respectively [21]. Additionally, the visibility of POPS can be affected by the choice of tomographic cut. Recent echocardiography evaluations have identified POPS in approximately 43% and 50% of patients using apical and parasternal windows, respectively [37]. However, ultrasound-based studies in this area used different cut plans for wall motion assessment and definitions of POPS, leading to variability in the results.

In summary, POPS can be predicted in about half of the patients, regardless of gender and type of CS. The prevalence of POPS is significantly influenced by various factors, including the timing of surgery and examination, the imaging technique used, and the definition applied.

**Table 1:** Characteristics of patients in previous studies. AVR: Aortic Valve Replacement; IVS: Interventricular Septum; CABG: Coronary Artery Bypass Graft; CPB: Cardiopulmonary Bypass; GBPS: Gated Blood Pool Scintigraphy; MVR/Rep: Mitral Valve Replacement/Repair; M-Mode: Motion-mode imaging; NMR: Nuclear Magnetic Resonance; POPS: Post-operative Paradoxical Septum; PTV: Parasternal Views; PW: Posterior Wall; RV: Right Ventricle; SPECT: Single Photon Emission Computed Tomography; TEE: Transoesophageal Echocardiogram; VHS: Valve Heart Surgery; VVI: Velocity Vector Imaging; 4CHV: Four-chamber Apical View.

Author/Year	N. TOT	Male (%)	Mean Age of Patient	Type of Surgery	% POPS *		Imaging other than TTE M-Mode and 2d	Explanation Theories
					<3 Months **	Follow-Up		
Burggraf, 1975 [2]	50	50	38	19 AVR 17 MVR 14 Other VHS	51 ^§^	15	-	Related to CPB
Righetti, 1977 [3]	40	77	57	40 CABG	57	20	Radionuclide angiography	Transient ischemic injury and exaggerated cardiac mobility due to pericardiotomy
Vignola, 1979 [4]	45	-	51	7 CABG 14 AVR 14 MVR 10 Others	53	-	GBPS	Related to CPB
Matsumoto, 1980 [5]	24	67	58	12 CABG 7 AVR 5 MVR	10	-	Intraoperative TEE	Exaggerated cardiac mobility due to pericardiotomy
Waggoner, 1982 [38]	17	56	56 ± 13	12 CABG 3 AVR 2 Others	60	-	Intraoperative direct-M-Mode post-operative 2D TTE	Exaggerated cardiac mobility due to pericardiotomy
Rubenson, 1982 [23]	20	90	62 ± 15	20 CABG	58	-	TTE 2D	-
Kerber, 1982 [32]	25	-	-	4 AVR 14 MVR 6 Others	56	28	TEE 2D	Exaggerated cardiac mobility due to limited RV free wall mobility
Gourdier, 1982 [30]	256	/	/	256 VHS	44	-	-	Exaggerated cardiac mobility
Force, 1983 [39]	20	20	59	17 CABG 2 AVR + CABG 1 AVR	68	-	TTE 2D (floating axis) radionuclide ventriculography	Exaggerated cardiac mobility
Akins, 1984 [40]	22	68	52	22 CABG	50	-	Resting GBPS or ventricular angiography	Related to CPB and/or myocardial preservation techniques.
Schroeder, 1985 [31]	324	/	/	110 CABG 214 HVS	69	14	-	Exaggerated cardiac mobility due to pericardiotomy
Schnittger, 1985 [41]	21	-	-	14 CABG 6 HSV 1 Other	76	-	Intraoperative direct M-Mode	Exaggerated cardiac mobility due to pericardiotomy
Feneley, 1987 [20]	16	87	52	15 CABG 1 Other	56	0	Intraoperative direct M-Mode post-operative TEE	Exaggerated cardiac mobility
De Nardo, 1989 [35]	34	88	55.2± 7.0	34 CABG	41 (Radionuclide angiocardiography) 29 (echocardiography)	-	Radionuclide angiocardiography	Exaggerated cardiac mobility due to pericardiotomy
van der Wall, 1990 [34]	12	75	41	12 AVR	-	92	Radionuclide angiography	Rigid ring of prosthesis limiting septal excursion
Lehmann, 1990 [21]	21	76	59.6 ± 9.6	18 CABG 2 HVS 1 Other	100 qualitatively 76 quantitatively	-	Intraoperative TEE	Related to CPB
Okada, 1992 [33]	16	100	59	16 CABG	100	100	Thallium-201 Scintigraphy Gated blood pool scan Tc 99m	Excluded ischemic injury
Wranne, 1993 [25]	19	52	54	4 CABG 4 CABG + HVS 6 MVR/Rep 3 AVR 2 Others	29 (before chest closure) 84 (after chest closure)	-	Intraoperative TEE	Recruitments of IVS to maintain RV global performance
Gigli, 1995 [42]	10	80	60 ± 9	10 CABG	50	-	TEE cyclic gray-level variation study	Excluded ischemic injury
Giubbini, 2004 [22]	82	86	67.8 ± 9.6	82 CABG	-	93	SPECT Tc 99	Excluded ischemic injury
Hedman, 2004 [43]	99	86	65 ± 9	99 CABG	-	96	-	Recruitments of IVS to maintain RV global performance
Toyoda, 2004 [44]	12	83	62 ± 11	12 CABG	75 (not specified the interval time from the intervention)		TDI	Exaggerated cardiac mobility
Reynolds, 2007 [9]	2979	-	-	1808 CABG 687 AVR 759 MVR/Rep 45 Others	40 (not specified the interval time from the intervention)		None	Related to the type of surgery and surgical approach
Joshi, 2008 [26]	23	73	64	23 CABG	-	100	NMR	Recruitments of IVS to maintain RV global performance
Roshanali, 2008 [36]	240	79	58.3 ± 11.3	240 CABG	-	97	TDI	Recruitments of IVS to maintain RV global performance
Choi, 2010 [45]	18	56	58 ± 12	18 CABG	-	56	NMR rest/stress	Exaggerated cardiac mobility due to pericardiotomy
Codreanu, 2011 [46]	18	100	67 ± 7	18 CABG	-	100	NMR high temporal resolution tissue phase mapping	Adhesion limiting the rotational motion of LV pushing IVS anteriorly during systole
Michaux, 2011 [47]	50		65 ±8 cohort A 61 ± 9 cohort B	50 CABG	32	-	TDI	No correlation with CPB
Kang, 2014 [24]	165	56	60 ± 13	59 CABG 99 VHS 7 Other	73	25	TOE-VVI	Related to subtle conduction disturbance
Moya Mur, 2018 [37]	30	60	69.9 ± 13.3	7 CABG 11 AVR 2 MVR 10 Other	50 in PTV 43 in 4CHV	-	TTE-STI	Exaggerated cardiac mobility due to limited RV free wall mobility

* The percentage refers to the actual cases studied that do not always correspond to the starting number of subjects, mainly during the follow-up. ** The early evaluation varies a lot among studies. Some studies performed early and late follow-ups within three months after the intervention. In this case, we reported the higher incidence observed in the period. ^§^ They performed the early follow-up within two months and the late beyond.

## 3. Normal and Paradoxical Interventricular Septum

**Figure 1:**
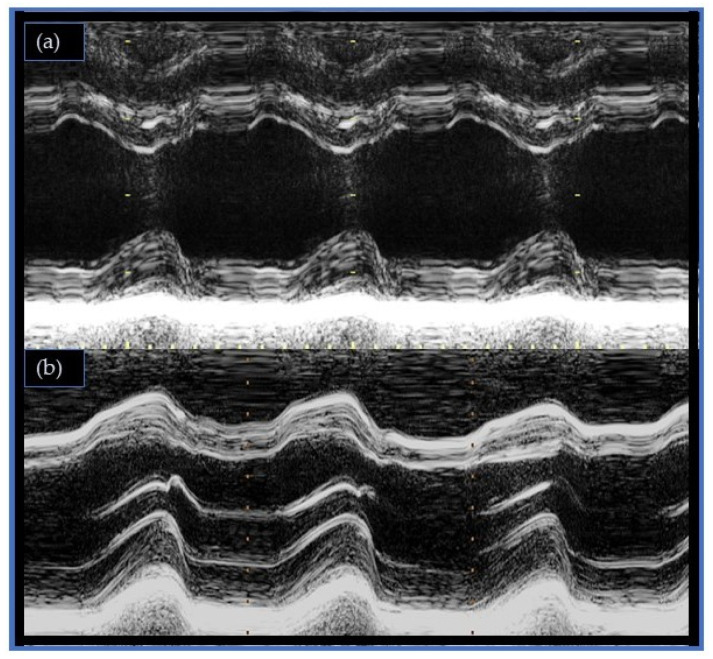
(**a**) Normal septal motion pattern (**b**) Postoperative paradoxical septal motion (Motion-mode imaging).

The IVS represents the keystone for interventricular coupling and the biventricular performance [27,48]. Unlike the other ventricular walls, IVS is directly exposed to both intraventricular pressures and influenced by the systolic and diastolic trans-ventricular gradients. Under normal conditions, it thickens during systole, increases its curvature, moves towards the LV center, and returns to its original position during diastole [49]. Breathing patterns can influence IVS motion during diastole, with inspiration displacing it posteriorly and expiration moving it in the opposite direction. This breathing-related effect on IVS motion is minimal under normal circumstances but can be more significant in pathological conditions [8,19]. When the IVS moves in the opposite direction to the physiological motion, it is called a “paradoxical” septum (Figure 1). A differential diagnosis is important for interpreting other paradoxical septa (Table 2) [50], but a comprehensive discussion of them is beyond the scope of this review.

## 4. The Septal Injury Theory

During the perioperative period, patients may experience complications such as type 5 acute myocardial infarction (MI) and procedure-related myocardial injury, particularly during cardiopulmonary bypass (CPB) [51,52,53]. Burggraf and Craige, in 1975, were the first to suggest that CPB-related myocardial injury may contribute to the development of POPS [2]. Studies conducted in the 70s and 80s confirmed a higher incidence of POPS when CPB was performed, with different possible explanations being speculated, such as transient CPB-related damage and graft-related coronary steal phenomenon [3,4,30,54]. It is important to note that in these studies, evidence of ischemia was primarily based on a reduction in wall thickening as observed in M-Mode. In many cases, there was no other evidence of myocardial injury.

However, recent literature has criticized the ischemic hypothesis, as there is no electrocardiographic or laboratory evidence supporting the myocardial damage required to cause such acute and localized kinetic alterations [21,25,30,32]. Moreover, the presence of POPS in patients undergoing uncomplicated CS with patent coronary arteries in the pre-surgical angiographic control makes the ischemic hypothesis unlikely [32,34]. Additionally, studies using 2D imaging found no significant reduction in septal systolic wall thickening (SSWT) in the POPS subgroup [24,35,37,41,45]. However, a stunning localized effect with minimal release of myocardial enzymes, such as in Takotsubo syndrome, should be considered [51]. Anyway, no differences in ventricular deformation pattern, perfusion, and late gadolinium enhancement (LGE) in the POPS group were reported [24,26,45,46].

There is also conflicting evidence on the role of CPB in causing damage during CS. A prospective study on 22 patients found that POPS incidence was significantly higher after uncomplicated on-pump CABG compared to off-pump CABG (*p* < 0.0005) [40]. Other studies also reported a higher incidence of POPS in on-pump CABG and found that POPS was independently associated with the CPB time and preoperative septal perfusion on multivariate analysis [9,55,56]. However, some studies have found no differences in postoperative septal motion patterns between the two cohorts [24]. In a recent study, Michaux et al. randomized 50 patients for on-pump vs. off-pump CABG and found no differences in POPS incidence after 3 months [47].

To summarize, myocardial ischemia is considered an unlikely cause of septal dyskinesia after uncomplicated CS and POPS seem to involve a dissociation between wall thickening and displacement.

## 5. The Timing

The temporal aspect of POPS is significant, as it occurs after CS. Typically, it is observed on transthoracic echocardiography (TTE) performed within a week after the procedure. 

Intraoperative imaging studies helped to define the timing and shed light on the underlying pathophysiological mechanism. Early studies using intraoperative M-Mode echocardiography from an anterior approach yielded intriguing results. In a 1982 study, 17 patients undergoing CS were examined before and after pericardiotomy and just before chest closure. Surprisingly, no patients exhibited ASM at the end of the operation, including those with preoperative paradoxical IVS on TTE. However, after a week, approximately 60% of patients showed POPS, with no significant changes in LV dimensions or function [38]. Similar findings were observed in a subsequent study involving a comparable population [41]. Feneley et al. reported normal IVS motion during all intraoperative stages in 16 patients undergoing uncomplicated CS. However, about 50% of a subgroup of patients exhibited POPS when assessed by transoesophageal echocardiography (TEE) within two hours after surgery [20]. These findings indicated that POPS developed early after chest closure, but the precise moment remained to be determined.

In a shift of perspective, Lehmann et al. employed intraoperative TEE in a cohort of 21 patients undergoing their first CS. They quantitatively assessed LV motion during various intraoperative steps, comparing them with the baseline. Interestingly, they observed a sudden onset of ASM and compensatory lateral hyperkinesis immediately after discontinuation of CPB in 76% of subjects. No significant changes were noted in regional or global ventricular kinetics during previous steps. This discovery demonstrated the intraoperative detectability of POPS, reinforcing the association with CPB while dismissing myocardial injury as a probable cause [21]. Similar partially overlapping observations were made by Wranne et al. using a similar intraoperative approach, where ASM appeared either after CPB discontinuation or soon after chest closure [25]. Other authors confirmed the intraoperative development of ASM [42].

In summary, the timing of POPS is crucial, occurring after CS and typically detectable on TTE within a week post-surgery. Intraoperative imaging studies have provided valuable insights, revealing the early development of POPS after chest closure or CPB discontinuation.

## 6. The Reference System

The choice of observation system significantly affects the assessment of postoperative septal motion. Contact-based imaging methods remove the relative motion between the heart and the probe, which could affect the detection of POPS. In line with this observation, a study by Waggoner et al. revealed that intraoperative evaluation did not show any alterations in patients with preoperative ASM, which was due to previous CS. In contrast, patients who had right-side overload showed paradoxical IVS until the atrial defect was corrected [38]. Reoperated patients with POPS have been less studied, but the presence of a true paradoxical IVS should be evident regardless of the imaging technique used or external factors [25]. In 1973, Miller et al. first described POPS in patients after uncomplicated MVR. Moreover, they found unexpectedly that patients with significant residual mitral or aortic regurgitation had normal septal motion. After correcting the LV volume overload, those patients paradoxically developed an ASM [1]. It seemed that after CS, the normal IVS motion could be reversed. Consequently, some authors have suggested excluding the IVS from postoperative LV kinetic evaluations.

The “floating system method” overcomes the limitations of M-mode imaging by superimposing traced endocardial end-diastolic and end-systolic 2D images using a defined intraventricular point of reference known as the “centroid”.

**Figure 2:**
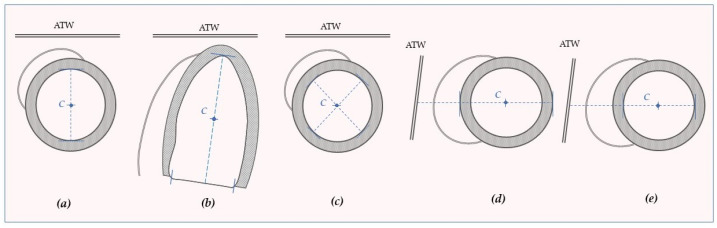
Schematic representation of left ventricular centroids used in the literature for post-operative septal motion assessment: (**a**) intermediate point between the IVS and the PW endocardium from short-axis view. (**b**) intermediate point between the apex and the mitral valve plane midpoint from the apical four-chamber view. (**c**) center of two perpendicular lines bisecting the cross-sectional area from a parasternal short-axis view. (**d**) the intermediate point between the IVS and the PW epicardium from a short-axis view. (**e**) intermediate point between the IVS and the PW endocardium from a short-axis view. ATW: Anterior Thoracic Wall; C: Centroid.

Various approaches have been employed (Figure 2). In their study, Waggoner et al. used the centroid (Figure 2a) to show a significant anteriorization of the LV after CS compared to unoperated healthy subjects. However, their method, which was similar to M-Mode imaging, did not provide information on the LV geometry [38]. To address this limitation, a more advanced approach was introduced, which defined both a center and an axis of reference (Figure 2b). Patients with POPS exhibited a notable anterior shift in the centroid, coupled with decreased septal kinesis and augmented kinesis of the lateral wall using an external reference. On the contrary, there were no noticeable differences in the kinetics of the LV walls when utilizing the floating system method [39].

Subsequent studies using TEE and NMR further supported the use of mobile reference systems. Intraoperative studies described the simultaneous appearance of wall kinetics anomalies and the significant increase in anteromedial translation of the centroid (Figure 2c) [21]. This finding was also corroborated by NMR in a subsequent study using a similar Waggoner’s approach after uncomplicated CABG (Figure 2d). They measured a significant increase in the postoperative systolic anterior displacement of the IVS, LV lateral wall, and LV centroid after intervention (*p* = 0.001) [26]. Other studies corroborated this evidence, showing decreased postoperative septal displacement in the ASM group, while SSWT remained unchanged (Figure 2e) [45].

**Figure 3:**
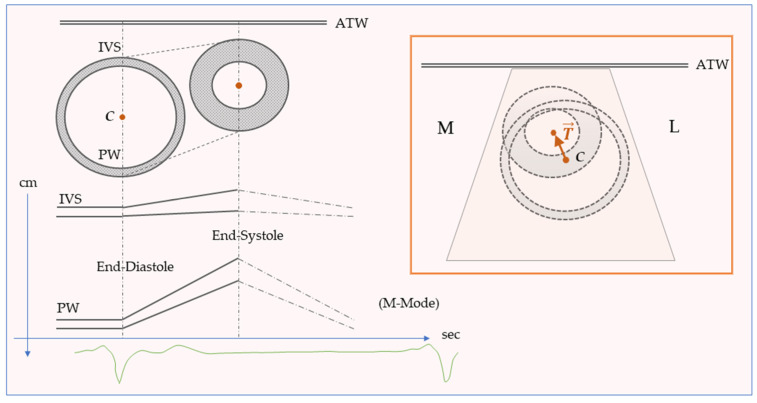
Schematic representation of the systolic left ventricular anterior translation movement (**above**) and corresponding mono-dimensional echocardiography pattern (**below**). In the orange box: schematic representation of the anterior-medial translation of the left ventricle from a parasternal-short axis view. The combined ventricular movement of anteromedial translation and radial contraction produces a hypokinetic septum and hyperkinetic posterior wall in the Motion-Mode scan. ATW: Anterior Thoracic Wall; C: Centroid; IVS: interventricular septum; L: Lateral side; M: Medial side; M-Mode: Motion-mode Imaging; PW: posterior wall; T: centroid translational vector.

In cases of uncomplicated CS, these findings suggest that LV experiences an increased postoperative anteromedial translational motion. The translation motion and wall thickening in the same range of values support the development of an appreciable optical effect explained by the composition of motions (Figure 3).

## 7. Curvature and Deformation

In addition to the floating system method, 2D imaging offers another way to evaluate kinetic independent of any translational motions. The LV has an approximately conical shape and physiologically maintains a concave shape throughout the cardiac cycle. When dealing with paradoxical IVS, such as in pulmonary hypertension, the curvature radius of the IVS increases [57]. However, studies have shown that the curvature of the LV and the LV’s eccentricity index remain unchanged after uncomplicated CS [20,21,32,58].

To obtain objective results and address the challenges posed by translation and traction motions when evaluating the postoperative LV regional kinetics, the study of LV curvature and a floating system have been used.

Additionally, tissue characterization and deformation evaluation techniques have emerged as valuable tools to differentiate between IVS function and mere displacement [15,22,59,60,61,62]. In this regard, Giubbini et al. retrospectively compared subjects with previous anterior MI (all with pre-operative hypokinetic IVS) and others with stable angina (with pre-operative normokinetic LV) after CABG. After the intervention, both cohorts expressed a similarly high incidence of ASM. However, only patients with previous MI showed a significant reduction in normalized septal thickening and perfusion (*p* < 0.0001) [22]. Similarly, one study found that patients with previous anterior MI had a reduction in strain parameters compared to those with stable angina after CABG, despite both groups having a similar rate of ASM. The CABG group also demonstrated significant anteromedial displacement of the LV, resulting in reduced tissue Doppler septal velocity when sampled from an apical approach or inverted when sampled from a parasternal approach [44]. Moreover, others have shown intact septal thickening and similar deformation parameters between cohorts with and without POPS, supporting the idea of a preserved septal function in both cases [37,46]. In a prospective study of 165 patients, LV global and regional peak circumferential strain and strain rate remained similar pre- and post-operatively. However, the POPS cohort showed significantly reduced radial systolic velocities of IVS. The authors ruled out the possibility of IVS injury based on intact septal thickening and similar deformation parameters between the groups [24]. While some authors suggested that the differences in segmental rotation velocities were due to friction to the anterior thoracic wall [46], others proposed that minor postoperative conduction disorders could be the reason for POPS [63]. However, the absence of intraventricular conduction delays on postoperative electrocardiograms casts doubt on this explanation [24]. Moreover, no correlation between LV dyssynchrony and previous CABG was observed in a large, heterogeneous cohort using SPECT or positron emission tomography (PET) [64].

The lack of alterations in LV geometry and function reported in various studies has shifted attention toward extrinsic factors as potential contributors to POPS. The exaggerated anterior motion of the LV during systole has been suggested as a possible mechanism, although the underlying cause of this translation motion is still subject to debate after many years of research.

## 8. The Role of the Pericardium

The pericardium plays a crucial role in stabilizing the heart and facilitating its physiological movements without friction. After CS, it was often left open, leading some researchers to suggest that the lack of pericardial integrity may contribute to postoperative exaggerated heart mobility [5,25,31,35,41,45]. In agreement with this assumption, patients who have had CS or those with CAP exhibit similar echocardiographic features, including ASM, excessive heart mobility, and increased PW displacement [17,65]. Consistently, the development of post-pericardiectomy ASM was described in [66]. However, the absence of intraoperative changes in septal kinetics after pericardiotomy has led to reevaluating the pericardium’s role [21,25,58]. Additionally, closure of the pericardium after CS does not appear to affect the development of ASM, as evidenced by a study by Lindqvist et al., which found no significant differences in bi-ventricular function and morphology during follow-up after pericardial repair at the end of AVS [67].

Other factors, such as the removal of the anterior mediastinal tissue, have been proposed as potential contributors to the development of exaggerated heart motion [39].

Over time, researchers have moved from thinking that excessive cardiac mobility was due to the pericardiotomy to believing that friction between the heart and surrounding tissues can lead to POPS.

## 9. The Right Ventricle: The Other Side of The Coin

It has been observed that RV longitudinal function (RVLF) tends to decrease after surgical procedures. There is an intriguing relationship between postoperative RV function adaptation, excessive heart motion, and POPS.

Kerber et al. first speculated on the role of RV in the POPS genesis. In their hypothesis, the RV attached to the anterior thoracic wall drags the entire heart anteriorly, contracting [32]. Friction between the heart and anterior mediastinum may explain the reduction of rotational and radial septal velocities described after uncomplicated CS [24,46]. Consistently, post-interventional systolic anteriorization of the heart has been observed using NMR [26]. Moreover, STI studies have demonstrated a postoperative shift of the ventricular longitudinal static reference point from the LV apex to the RV-free wall [37]. This resulted in a postoperative reduction of RV basal longitudinal velocity, strain, and displacement while bi-ventricular global systolic function remained stable [37].

The return to normal values of tricuspid annular plane systolic excursion (TAPSE) after adhesiolysis in patients who underwent a second cardiac intervention supports these findings [25].

However, the traction of the heart due to adhesions and containment of the anterior thoracic wall fails to explain the intraoperative ASM development [21].

In 1993, Wranne et al. demonstrated that RVLF impairment and POPS occurred during the same intraoperative phases [25]. Several subsequent studies reported similar findings, leading to the hypothesis that postoperative reduced RVLF may lead to compensatory movement of the IVS to maintain stable ventricular function [25,28,58]. This is supported by studies that show a significant correlation between septal systolic anterior motion and reduced TAPSE (r = 0.60; *p* < 0.001) [67], as well as between septal systolic anterior motion and RV ejection fraction (r = 0.47; *p* = 0.023) [26]. Moreover, patients with normokinetic IVS showed preserved RVLF [36]. Some experimental models with a dysfunctional RV-free wall showed that IVS compensates for RV-impaired systolic function [68].

The RV contraction comprises three mechanisms: the base-apical displacement (the most important in the normal setting, contributing to up to two-thirds of the output), the radial contraction, and the traction of the free wall by the twisting-LV [48,69]. Postoperatively, the RV shows a peculiar functional adaptation consisting of reduced longitudinal displacement with increased radial wall displacement and unaltered global function [28,37,45,58,70]. Some authors have also reported a relative RV distension [4,71]. Postoperative changes in RV morphology and function occur rapidly but may persist for a prolonged period [36,43].

While there are commonalities between excessive heart motion, POPS, and RVLF impairment, they are not always interconnected, and the degree of RV dysfunction required for POPS development remains unclear. The hypothesis of adaptive IVS compensation for RV function aligns with the intraoperative development of POPS and excessive heart motion and the maintenance of RV global function [72].

## 10. A New Heuristic Hypothesis for POPS

The postoperative kinetic pattern of the LV characterized by anteroseptal hypo- and posterolateral hyperkinesis can be interpreted in different ways. One possibility is that it represents septal dysfunction compensated by PW hyperkinesis, resulting in an unchanged LVEF. However, this pattern is not consistent with the nature of injury-related postoperative complications, as there is a lack of significant markers of injury and normal ventricular perfusion and wall thickening [22,24,35,37,46]. Another interpretation is that the combined systolic displacement of the entire heart and LV walls produces the observed motion pattern when viewed from a fixed observational system like TTE. This scheme may account for the apparent discrepancy between LV function and motion. The anterior displacement of the centroid and PW associated with an unchanged SSWT supported this view [20,21,38,39]. The septal kinetics abnormalities observed in patients with POPS may be reversible during a second CS and tend to resolve over time [25]. Moreover, the return to a normal centroid displacement range during follow-up also reinforces this idea [38,39]. However, despite knowing when it manifests during surgery, when and how it resolves remains debated. In an uncomplicated setting, POPS does not seem to have significant clinical consequences, and changes in pharmacological management are not currently recommended since data on the negative clinical impact of POPS are lacking [36,43,73].

Impaired RVLF after CS has been proposed as a possible cause of POPS. Both POPS and RVLF impairment develop intraoperatively and can normalize over time [25,26,28,36]. Postoperatively, the RV motion’s pattern and geometry change, while LV morphology does not, leading to speculation that the RV plays a central role in the biventricular motion pattern changes. The POPS may represent a compensative mechanism for the impaired RVLF [25,26,58,67]. The exact trigger for RVLF impairment and its transmission to the LV is not fully understood, but it appears to be linked to the surgery itself, independent of other variables [74]. Biventricular motion patterns change rapidly after CS without significant ventricular dysfunction. The possibility of an acute postoperative myopathic state of the endocardial fibers has been considered. However, several studies have demonstrated the preservation of ventricular contractility using strain parameters [28,37,46,67]. Moreover, most reports indicate that global ventricular function and clinical status remain unchanged or improve after CS despite the impaired RVLF [26,28,36,43,58,67]. These findings challenge the idea of compensatory mechanisms due to abrupt functional asymmetry between the ventricles.

**Figure 4:**
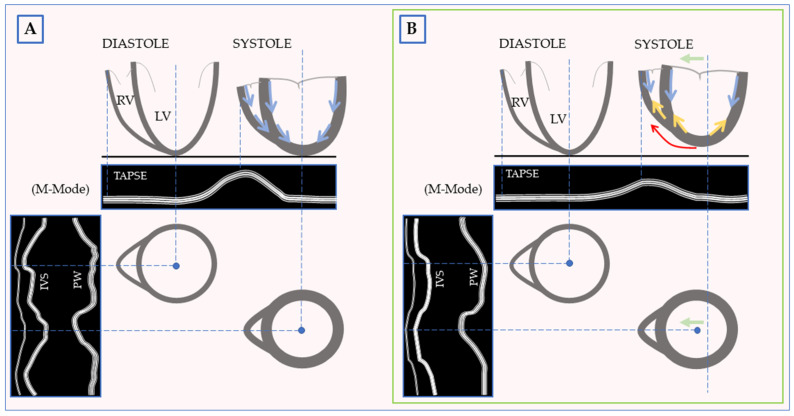
The connection between postoperative paradoxical septum (POPS) and right ventricular longitudinal function (RVLF). (**A**) Schematic representation of normal bi-ventricular motion. (**B**) Post-operative bi-ventricular motion according to our hypothesis. After surgery, the reduced relative apical fixation produces an anterior heart displacement (green arrow) accounting for POPS and a reduced basal-apical tricuspid annular displacement. An accentuated rotation (red arrow) could contribute to the post-operative reduction of TAPSE and septal MAPSE. The blue and yellow arrows indicate the prevailing longitudinal direction displacement of the left ventricle during systole. IVS: interventricular septum; M-Mode: Motion-mode Imaging; LV: Left Ventricle; MAPSE: mitral annular plane systolic excursion; PW: posterior wall; RV: Right Ventricle; TAPSE: tricuspid annular plane systolic excursion.

A heuristic geometric explanation for the biventricular kinetic pattern changes is proposed. The contraction of the LV involves twisting, shortening, and thickening motion, with myocardial fibers arranged in a specific pattern moving from one to another coplanar point on the cardiac fibrous skeleton [27,48,49]. During contraction, they move centripetally towards the base and the central axis. Yet, there is a prevailing movement of the base towards the apex due to the relative fixation of the phrenic cardiac surface and the apex cordis. We wonder how the kinetics of a normal heart hanging on its hilum vary. It could be different than a heart in situ, despite maintaining normal function. In the described condition, the medial ventricular segments remain longitudinally static, and the contraction of the RV-free wall drags the LV anteriorly, eliminating the need for any friction mechanism (Figure 4). The postoperative increase in anteromedial LV rotation supports our conjecture [20,39]. This mechanism may explain the intraoperative development and its correlation with the CPB of both the POPS and the RVLF impairment. Additionally, it does not indicate any functional asymmetry between the ventricles.

Removal of anterior mediastinal tissue and the CBP-related impairment of the right atrium may also contribute to this mechanism [38,39,67].

After chest closure, friction and adhesions between the heart and thoracic tissues could further enhance this pattern by fixing the mid-basal sternal cardiac surface [32,37,46].

## 11. Conclusions

In conclusion, after uncomplicated CS, there are changes in the bi-ventricular kinetic pattern without negative clinical impact, maintaining preserved systolic function. Various theories intended to explain this phenomenon. Selective IVS damage and significant conduction disturbances are considered unlikely causes of POPS. Currently, the most widely accepted hypothesis is that a combination of left ventricular contraction and anterior translation is responsible for increased cardiac motility. Although it was initially believed that the absence of pericardial constrictions was the primary cause, recent evidence suggests that it may increase friction with the surrounding mediastinum. Early studies focused primarily on the LV and interpreted its motion as that of the entire heart. However, the RV appears more static postoperatively with impaired longitudinal performance. Some researchers have suggested that POPS compensates for impaired RVLF, but the idea of significant functional asymmetry between the ventricles is criticized. We conjecture that postoperative bi-ventricular kinetic changes could be related to a shift in anchor ventricular points from the apex toward the anteromedial basal portion. Intraoperatively, anterior mediastinal tissue removal and CPB may contribute, while adhesions with the anterior thorax appear to be the main determinant after chest closure. According to this perspective, the paradoxical motion pattern may express normal biventricular kinetics in the postoperative period.

## Figures and Tables

**Table 2:** Characteristics and types of abnormal septal motion. IV: Intraventricular; IVS: Interventricular Septum; LBBB: Left Bundle Branch Block; LV: Left Ventricle; POPS: post-operative paradoxical septum; RV: Right Ventricle; (*) could also be diastolic (**) could be systolic and/or diastolic.

Characteristics of Abnormal Septal Motion	Common Causes of Abnormal Septal Motion						
	POPS	LBBB/RV Pacing Rhythm	Ischemia	Rv Pressure/ Volume Overload	Constrictive Pericarditis	Pericardial Tamponade	Obstructive Pulmonary Diseases/Mechanical Ventilation
Systolic	+	+	+ (*)	+ (**)	−	−	−
Normal Iv Conduction	+	−	+	+	+	+	+
Preserved Ivs Perfusion	+	+	−	+	+	+	+
Normal Ivs Metabolism	+	+	−	+	+	+	+
Normal Lv Geometry	+	−	−	−	−	−	−
Normal Lv Global Systolic Function	+	+	+/−	+/−	+/−	+/−	+/−
Respirophasic Motion	−	−	−	−	+	+	+
Stress Related	−	+/−	+/−	−	−	−	−

## Data Availability

Not applicable.
